# Correction to ‘The role of diet in multiple sclerosis onset and course: Results from a nationwide retrospective birth‐year cohort’

**DOI:** 10.1002/acn3.51904

**Published:** 2023-12-07

In the originally published version of the article, Table 3 on page 1275 lacks of differentiation between multivariable ‘a’ and ‘b’ under each brain volume, this has been corrected as follows.

**Table 3:** Multivariable associations between individual dietary components, overall diet quality and MRI volumetric measures.

Variables	MS, *n*	NBV		NWMV		NCGMV		NDGMV	
		Multivariable a	Multivariable b	Multivariable a	Multivariable b	Multivariable a	Multivariable b	Multivariable a	Multivariable b
Consumption at age 50 serves/week		Unst. B (95% CI)	Unst. B (95% CI)	Unst. B (95% CI)	Unst. B (95% CI)	Unst. B (95% CI)	Unst. B (95% CI)	Unst. B (95%CI)	Unst. B (95%CI)
Fruit									
0–5	126	1	1	1	1	1	1	1	1
5–7	91	21.6 (−4.9–48.0)	21.1 (−5.45–47.7)	0.3 (−14.8–15.3)	0.7 (−14.3–15.7)	18.8 (1.2–36.4)*	17.8 (0.3–35.3)*	3.0 (1.2–4.8)**	3.0 (1.2–4.8)***
≥7.0	127	19.5 (−4.7–43.6)	18.0 (−6.8–42.7)	4.4 (−9.36–18.1)	4.9 (−9.1–18.8)	14.0 (−2.0–30.0)	11.8 (−4.4–28.1)	1.4 (−0.2–3.0)	1.5 (−0.1–3.2)
P for linear trend		0.126	0.169	0.518	0.481	0.106	0.179	0.137	0.101
Continuous		3.5 (−0.4–7.4)	3.3 (−0.8–7.2)	0.2 (−2.00–2.5)	0.4 (−1.9–2.6)	3.0 (0.4–5.6)*	2.7 (0.04–5.3)*	0.3 (−0.02–0.5)	0.3 (0.01–0.6)*
Vegetable									
0–6	143	1	1	1	1	1	1	1	1
6–7	**91**	29.5 (4.0–55.0)*	31.2 (5.5–57.0)*	14.7 (0.2–29.1)*	13.1 (−1.5–27.6)	13.6 (−3.5–30.7)	17.0 (−0.1–34.0)	1.9 (0.1–3.6)*	1.9 (0.1–3.7)*
≥7	127	12.8 (−10.6–36.2)	12.0 (−11.6–35.5)	−1.0 (−14.3–12.2)	−1.4 (−14.6–11.9)	13.2 (−2.5–28.9)	12.7 (−2.8–28.3)	1.0 (−0.6–2.6)	1.0 (−0.6–2.7)
P for linear trend		0.253	0.278	0.950	0.910	0.092	0.096	0.197	0.185
Continuous		8.0 (−0.7–16.7)	7.8 (−1.00–16.6)	−0.02 (−5.0–4.7)	−0.04 (−5.0–4.9)	7.6 (1.8–13.4)*	7.4 (1.6–13.1)*	0.5 (−0.1–1.1)	0.6 (−0.04–1.2)
Oily fish									
0	97	1	1	1	1	1	1	1	1
1–2	162	−2.9 (−27.5–21.6)	−4.0 (−28.6–20.7)	8.1 (−5.8–22.0)	8.5 (−5.3–22.4)	−9.0 (−25.4–7.4)	−10.5 (−26.7–5.7)	−1.4 (−3.1–0.2)	−1.4 (−3.1–0.3)
≥2	101	−14.1 (−41.7–13.5)	−16.6 (−44.7–11.5)	0.5 (−15.1–16.1)	1.1 (−14.7–16.8)	−12.7 (−31.2–5.7)	−15.8 (−34.2–2.7)	−1.0 (−2.8–0.9)	−0.9 (−2.8–1.0)
P for linear trend		0.310	0.240	0.973	0.917	0.177	0.094	0.335	0.381
Continuous		−4.1 (−14.4–6.2)	−5.1 (−15.7–5.4)	1.7 (−4.5–7.2)	1.6 (−4.4–7.5)	−4.8 (−11.6–2.1)	−6.0 (−12.9–0.9)	−0.3 (−1.0–0.4)	−0.3 (−1.0–0.4)
Red meat									
0	88	1	1	1	1	1	1	1	1
1–3	160	−8.2 (−33.5–17.0)	−7.2 (−32.7–18.3)	−1.21 (−15.5–13.1)	−1.1 (−15.5–13.2)	−6.4 (−23.3–10.5)	−5.4 (−22.2–11.4)	0.3 (−1.5–2.0)	0.2 (−1.5–2.0)
≥3	112	−8.7 (−36.6–19.2)	−7.9 (−36.0–20.2)	−0.8 (−16.7–15.0)	−1.9 (−17.7–13.9)	−7.2 (−25.8–11.5)	−5.3 (−23.8–13.3)	0.2 (−1.7–2.1)	0.2 (−1.7–2.1)
P for linear trend		0.557	0.593	0.925	0.811	0.466	0.597	0.854	0.884
Continuous		−2.19 (−8.7–4.3)	−2.1 (−8.6–4.5)	−0.4 (−4.1–3.3)	−0.7 (−4.4–3.0)	−1.6 (−5.9–2.8)	−1.2 (−5.6–3.1)	−0.01 (−0.5–0.4)	−0.01 (−0.5–0.4)
Whole grain bread									
0–5	104	1	1	1	1	1	1	1	1
5–7	79	−9.88 (−37.2–17.4)	−9.3 (−36.71–18.2)	−5.3 (−20.7–10.2)	−5.7 (−21.1–9.7)	−4.2 (−22.5–14.1)	−3.1 (−21.2–15.0)	−0.4 (−2.3–1.2)	−0.5 (−2.3–1.4)
≥7	177	−3.66 (−27.0–19.7)	−5.00 (−28.61–18.7)	−4.5 (−17.7–8.8)	−5.0 (−18.3–8.3)	1.2 (−14.4–16.8)	0.5 (−15.1–16.1)	−0.5 (−2.1–1.1)	−0.4 (−2.0–1.2)
P for linear trend		0.811	0.715	0.534	0.481	0.825	0.992	0.578	0.630
Continuous		−1.4 (−12.0–10.2)	−2.2 (−14.0–9.6)	−2.1 (−8.6–4.5)	−2.4 (−9.0–4.3)	0.9 (−6.9–8.6)	0.4 (−7.4–8.2)	−0.2 (−1.0–0.6)	−0.2 (−1.0–0.6)
Snacks, candy and fast‐food									
0–6	93	1	1	1	1	1	1	1	1
6–12	106	2.7 (−24.3–29.6)	2.07 (−25.1–29.3)	−1.7 (−17.1–13.6)	−2.8 (−18.2–12.6)	3.4 (−14.5–21.3)	3.9 (−13.9–21.6)	1.0 (−0.8–2.9)	1.1 (−0.8–2.9)
≥12	160	17.6 (−7.5–42.6)	17.59 (−7.7–42.9)	−2.0 (−16.3–12.3)	−3.6 (−17.9–10.7)	17.5 (0.8–34.1)*	19.1 (2.6–35.6)*	1.8 (0.1–3.5)*	1.8 (0.1–3.5)*
P for linear trend		0.134	0.134	0.793	0.633	**0.027**	**0.015**	**0.044**	**0.043**
Continuous		1.2 (−1.2–3.7)	1.25 (−1.2–3.7)	−0.2 (−1.6–1.1)	−0.4 (−1.8–1.0)	1.3 (−0.3–2.9)	1.5 (−0.1–3.1)	0.2 (0.01–0.3)*	0.2 (0.01–0.3)*
Overall diet quality score									
0–4	98	1	1	1	1	1	1	1	1
4–7	120	13.8 (−11.6–39.1)	12.6 (−12.9–38.1)	1.67 (−12.7–16.0)	2.6 (−11.7–17.0)	11.7 (−5.2–28.5)	9.5 (−7.3–26.2)	0.9 (−0.8–2.7)	1.0 (−0.8–2.7)
≥6.5	141	−3.9 (−29.4–21.6)	−6.6 (−32.7–19.5)	−3.7 (−18.1–10.7)	−2.6 (−17.3–12.1)	0.7 (−16.2–17.7)	−3.2 (−20.3–13.9)	−0.5 (−2.3–1.2)	−0.5 (−2.2–1.3)
P for linear trend		0.707	0.569	0.594	0.704	0.991	0.664	0.497	0.567
Continuous		0.6 (−4.2–5.4)	0.07 (−4.9–5.0)	0.18 (−2.5–2.88)	0.4 (−2.4–3.1)	0.6 (−2.6–3.8)	−0.1 (−3.4–3.1)	−0.5 (−0.4–0.3)	−0.02 (−0.4–0.3)

Variables		NThalV		NCbV		MUCCA		LV	
		Multivariable a	Multivariable b	Multivariable a	Multivariable b	Multivariable a	Multivariable b	Multivariable a	Multivariable b
Consumption at age 50 serves/week		Unst. B (95% CI)	Unst. B (95% CI)	Unst. B (95% CI)	Unst. B (95% CI)	Unst. B (95% CI)	Unst. B (95% CI)	Unst. B (95% CI)	Unst. B (95% CI)
Fruit									
0–5	126	1	1	1	1	1	1	1	1
5–7	91	0.7 (0.04–1.4)*	0.7 (0.06–1.4)*	5.3 (0.7–9.9)*	5.1 (0.5–9.7)*	4.7 (0.5–8.8)*	4.7 (0.5–8.9)*	−0.1 (−0.3–0.2)	−0.1 (−0.3–0.2)
≥7.0	127	0.4 (−0.2–1.0)	0.4 (−0.2–1.1)	2.9 (−1.2–7.1)	2.6 (−1.7–6.8)	5.4 (1.6–9.3)**	5.5 (1.6–9.4)**	−0.1 (−0.3–0.2)	−0.1 (−0.3–0.2)
P for linear trend		0.230	0.202	0.223	0.287	**0.007**	**0.007**	0.613	0.612
Continuous		0.1 (−0.02–0.2)	0.1 (−0.01–0.2)	0.5 (−0.2–1.2)	0.6 (−0.2–1.2)	0.8 (0.1–1.4)*	0.8 (0.5–1.4)*	−0.01 (−0.1–0.03)	−0.01 (−0.1–0.03)
Vegetable									
0–6	143	1	1	1	1	1	1	1	1
6–7	**91**	0.7 (0.03–1.2)*	0.6 (−0.05–1.2)*	0.9 (−3.7–5.4)	1.4 (−3.1–6.0)	1.7 (−2.5–5.8)	1.6 (−2.6–5.8)	−0.3 (−0.5–0.01)*	−0.3 (−0.5–0.01)*
≥7	127	0.3 (−0.3–0.9)	0.3 (−0.3–0.9)	0.8 (−3.3–5.0)	0.8 (−3.4–4.9)	0.03 (−3.8–3.8)	−0.1 (−3.9–3.8)	−0.2 (−0.4–0.03)	−0.2 (−0.4–0.03)
P for linear trend		0.243	0.258	0.686	0.697	0.960	0.996	0.072	0.073
Continuous		0.2 (−0.1–0.4)	0.2 (−0.1–0.4)	0.8 (−0.7–2.4)	0.8 (−0.7–2.3)	0.2 (−1.3–1.6)	0.1 (−1.3–1.6)	−0.1 (−0.2–0.00)*	−0.1 (−0.2–0.00)*
Oily fish									
0	97	1	1			1	1	1	1
1–2	162	−0.3 (−0.9–0.3)	−0.3 (−0.6–0.7)	−1.8 (−6.1–2.5)	−2.0 (−6.3–2.3)	−1.4 (−5.3–2.6)	−1.4 (−5.4–2.5)	−0.002 (−0.2–0.2)	−0.002 (−0.2–0.2)
≥2	101	−0.3 (−1.0–0.5)	−0.2 (−1.0–0.4)	−0.4 (−5.2–4.0)	−0.8 (−5.7–4.1)	−2.6 (−7.1–1.8)	−2.8 (−7.2–1.7)	0.1 (−0.2–0.4)	0.1 (−0.2–0.4)
P for linear trend		0.479	0.381	0.893	0.759	0.243	0.218	0.409	0.401
Continuous		−0.1 (−0.3–0.2)	−0.1 (−0.3–0.2)	−0.2 (−2.0–1.6)	−0.4 (−2.0–1.4)	−1.4 (−3.0–0.3)	−1.5 (−3.2–0.18)	0.04 (−0.1–0.1)	0.04 (−0.1–0.1)
Red meat									
0	88	1	1	1	1	1	1	1	1
1–3	160	0.00 (−0.7–0.7)	0.00 (−0.7–0.7)	2.4 (−2.1–6.8)	2.5 (−1.9–6.9)	5.0 (1.0–9.0)*	5.1 (1.1–9.2)*	0.1 (−0.1–0.4)	0.1 (−0.1–0.4)
≥3	112	−0.3 (−1.0–0.5)	−0.3 (−1.0–0.5)	0.9 (−4.0–5.7)	1.2 (−3.7–6.1)	3.7 (−0.7–8.2)	3.7 (−0.7–8.2)	0.3 (0.04–0.6)*	0.3 (0.04–0.6)*
P for linear trend		0.466	0.383	0.794	0.383	0.134	0.138	**0.022**	**0.023**
Continuous		−0.10 (−0.3–0.1)	−0.10 (−0.3–0.1)	−0.2 (−1.4–0.9)	−0.2 (−1.3–0.9)	0.7 (−0.3–1.8)	0.7 (−0.3–1.8)	0.1 (0.003–0.1)*	0.1 (0.003–0.1)*
Whole grain bread									
0–5	104	1	1	1	1	1	1	1	1
5–7	79	−0.2 (−0.9–0.5)	−0.2 (−0.9–0.5)	−0.04 (−4.8–4.7)	−0.1 (−4.6–4.9)	−2.7 (−7.0–1.7)	−2.7 (−7.1–1.7)	0.1 (−0.2–0.4)	0.1 (−0.2–0.4)
≥7	177	−0.1 (−0.7–0.5)	−0.1 (−0.7–0.5)	0.02 (−4.1–4.1)	0.1 (−4.2–4.0)	0.5 (−3.2–4.3)	0.5 (−3.3–4.2)	−0.03 (−0.3–0.2)	−0.03 (−0.3–0.2)
P for linear trend		0.806	0.753	0.990	0.963	0.656	0.716	0.720	0.723
Continuous		−0.04 (−0.3–0.3)	−0.05 (−0.4–0.3)	0.1 (−2.0–2.0)	0.1 (−2.1–2.0)	0.4 (−1.4–2.3)	0.4 (−1.5–2.2)	−0.02 (−0.1–0.09)	−0.02 (−0.1–0.09)
Snacks, candy and fast‐food									
0–6	93	1	1	1	1	1	1	1	1
6–12	106	0.5 (−0.2–1.2)	0.5 (−0.2–1.2)	3.9 (−0.8–8.6)	4.1 (−0.6–8.7)	3.2 (−1.1–7.6)	3.1 (−1.3–7.5)	−0.02 (−0.3–0.2)	−0.02 (−0.3–0.2)
≥12	160	0.6 (−0.1–1.2)	0.5 (−0.1–1.2)	6.2 (1.9–10.5)**	6.2 (2.2–10.9)**	2.9 (−1.1–6.9)	2.8 (−1.27–6.9)	0.01 (−0.2–0.3)	0.01 (−0.2–0.3)
P for linear trend		0.112	0.161	**0.006**	**0.003**	0.206	0.231	0.886	0.883
Continuous		0.1 (−0.01–0.1)	0.1 (−0.01–0.1)	0.7 (0.3–1.1)**	0.7 (0.3–1.2)***	0.2 (−0.2–0.6)	0.2 (−0.2–0.6)	−0.001 (−0.2–0.2)	−0.001 (−0.2–0.2)
Overall diet quality score									
0–4	98	1	1	1	1	1	1	1	1
4–7	120	0.3 (−0.4–0.9)	0.3 (−0.4–1.0)	3.0 (−1.4–7.4)	2.5 (−1.9–6.9)	−1.2 (−5.2–2.9)	−1.2 (5.3–2.9)	−0.3 (−0.5–−0.02)*	−0.3 (−0.5–−0.02)*
≥6.5	141	−0.1 (−0.7–0.6)	−0.1 (−0.7–0.6)	−1.7 (6.1–2.7)	−2.5 (7.0–1.9)	−0.2 (−4.3–3.9)	−0.3 (−4.5–3.9)	−0.2 (0.4–0.06)	−0.19 (−0.4–0.1)
P for linear trend		0.791	0.866	0.390	0.232	0.940	0.903	0.162	0.160
Continuous		0.02 (−0.1–0.1)	0.02 (−0.1–0.2)	−0.2 (−1.1–0.6)	−0.4 (−1.2–0.5)	−0.1 (−0.8–0.7)	−0.1 (−0.9–0.7)	−0.04 (−0.1–0.01)	−0.04 (−0.1–0.02)

*Note:* Bold values denote statistical significance at the *p* < 0.05 level, *p‐value < 0.05, **p‐value < 0.01, ***p‐value < 0.001.

Multivariable a: including the following covariates: sex, sun exposure at age 50, smoking status, disease duration since onset, disease modifying therapy duration, onset type, education.

Multivariable b: including the following covariates: sex, sun exposure at age 50, smoking status, disease duration since onset, disease modifying therapy duration, onset type, education, body mass index (BMI) and physical activity at age 50.

We apologize for this error.

Loonstra FC, de Ruiter LRJ, Schoonheim MM, et al. The role of diet in multiple sclerosis onset and course: results from a nationwide retrospective birth‐year cohort. Ann Clin Transl Neurol. 2023;10:1268‐1283. https://doi.org/10.1002/acn3.51788

